# Characterization of the complete chloroplast genome of *Plumeria rubra* cv. Acutifolia (Apocynaceae)

**DOI:** 10.1080/23802359.2020.1721023

**Published:** 2020-01-29

**Authors:** Dong-Lan Wang, Ying-Ying Liu, Dan Tian, Li-Ying Yu, Ling-Jian Gui

**Affiliations:** a Guangxi Botanical Garden of Medicinal Plants, Nanning, Guangxi, China;; b Tongren Polytechnic College, Tongren, Guizhou, China

**Keywords:** Apocynaceae, chloroplast genome, phylogenetic analysis

## Abstract

*Plumeria rubra* cv. Acutifolia is a widely planted landscape tree in the subtropics. In this study, the complete chloroplast genome of *P. rubra* cv. Acutifolia was determined through Illumina sequencing method. The complete chloroplast genome has a length of 153,912 bp, containing a small single-copy region (18,036 bp), a large single-copy region (84,852 bp), and a pair of IR regions (25,512 bp). The chloroplast genome possesses 130 genes, including 85 CDS, 37 tRNA genes and 8 rRNA genes. *P. rubra* cv. Acutifolia exhibited the closest relationship with *P. cubensis* in phylogenetic analysis.

*Plumeria rubra* L. cv. Acutifolia (Apocynaceae) is a deciduous shrub endemic to the new world (Haber 1984). It is a beautiful landscape tree with yellow and white flowers, loved by the people of China and Southeast Asian countries, which has been widely planted in the tropical and subtropical regions (Aguoru et al. 2015). In the traditional medical theories, *P. rubra* cv. Acutifolia has the effect of clearing away heat and toxic material (Hamburger et al. 1991; SATCM 1999). Chemical composition analysis showed that the flower contained volatile oil (Xiao et al. 2011), and had the potential to extract essential oil. With the aim to retrieve valuable chloroplast molecular markers and SSRs for research of conservation biology, we assembled and analyzed the complete chloroplast genome of *P. rubra* cv. Acutifolia based on the next-generation sequencing method.

The fresh leaves of *P. rubra* cv. Acutifolia were collected from Guangxi Botanical Garden of Medicinal Plants (22°51′N, 108°22′E) and the voucher specimen lyy03 was stored in the herbarium of Guangxi Botanical Garden of Medicinal Plants (GXMG). Sequencing was done on Illumina Hiseq-2500 platform to produce 150 bp paired-end reads. The clean data was assembled via NOVOPlasty (Dierckxsens et al. 2017). The complete genome was annotated in PGA-master (Qu et al. 2019), taking *Amborella trichopoda* (GenBank number AJ506156) and related species as references, combined with manual correction in Geneious 10.2 (Kearse et al. 2012). The complete genome was uploaded to GenBank with accession number MN812495. For phylogenetic analysis of the plastid, we selected other 15 chloroplast genomes of Apocynaceae from the National Center for Biotechnology Information (NCBI). 57 shared CDS were extracted from the sequences and aligned by MAFFT (Katoh et al. 2002). A maximum likelihood (ML) tree was constructed by Mega 7 (Kumar et al. 2016) with 500 bootstrap.

The complete chloroplast genome of *P. rubra* cv. Acutifolia shows a quadripartite structure with a length of 153,912 bp, containing a small single-copy (SSC) region (18,036 bp), a large single-copy (LSC) region (84,852 bp), and a pair of inverted repeat (IR) regions (25,512 bp). One hundred thirty genes were determined, including 85 CDS, 37 tRNA genes, and 8 rRNA genes. The total GC content of complete chloroplast genome was 37.9%. *P. rubra* cv. Acutifolia exhibited the closest relationship with *P. cubensis* in phylogenetic analysis (Figure 1). The results will provide useful information for biological and conservation research.

**Figure 1.**
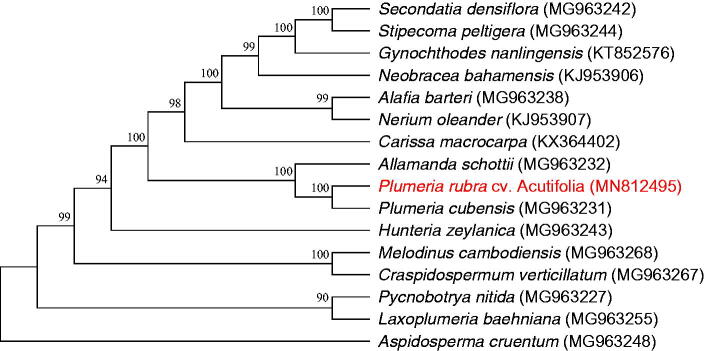
Maximum likelihood (ML) tree reconstruction of 16 taxa in Apocynaceae based on 57 shared CDS in the chloroplast genomes. Numbers above the branches are the bootstrap values.
